# Acceptability and Willingness to Pay for a Meal Kit Program for African American Families with Low Income: A Pilot Study

**DOI:** 10.3390/nu13082881

**Published:** 2021-08-21

**Authors:** Kaley Carman, Lauren H. Sweeney, Lisa A. House, Anne E. Mathews, Karla P. Shelnutt

**Affiliations:** 1 Department of Family, Youth and Community Sciences, University of Florida, Gainesville, FL 32611, USA; kmialki@ufl.edu; 2 Food Science and Human Nutrition Department, University of Florida, Gainesville, FL 32611, USA; lheadrick@ufl.edu (L.H.S.); anne.mathews@ufl.edu (A.E.M.); 3 Food and Resource Economics Department, University of Florida, Gainesville, FL 32611, USA; lahouse@ufl.edu

**Keywords:** meal kit, nutrition, low income, African American, cooking, food security, affordability, acceptability, home cooking

## Abstract

Food insecurity is a persistent issue among individuals with low income and is associated with various nutrition- and health-related consequences. Creative approaches to increasing food access should be investigated as possible solutions. Meal kits, which are boxes or bags of fresh and shelf-stable ingredients for one or more meals, along with a step-by-step recipe showing how to cook each meal at home, may serve as a creative solution. Meal kits have historically been marketed to higher-income demographics. The purpose of this pilot study was to investigate the utilization, acceptability, and willingness to pay for a healthy meal kit program among African American main food preparers with children and low income (n = 36). Participants received a healthy meal kit with three recipes and ingredients, a cooking incentive, and a nutrition handout weekly for six weeks. Data were collected on participants’ use, acceptability, and willingness to pay for the meal kits and analyzed using descriptive statistics. The intervention was highly utilized, and participants reported high acceptability ratings for most recipes. After the intervention, participants were willing to pay $88.61 ± 47.47 for a meal kit with three meals, each with four portions, which was higher than indicated at baseline and similar to the cost to produce the kits. Meal kits may offer a creative solution to improving food access if affordable for families with low income.

## 1. Introduction

Food insecurity is a persistent problem in the United States, with an estimated 10.5% of households in the country experiencing food insecurity in 2019 [1]. Households with children, single-parent households, Black and Hispanic households, and households with low income experience food insecurity at higher rates [2]. Feeding America estimated that food insecurity rates rose during the COVID-19 pandemic to 15.6%, and that it continued to disproportionately affect racial/ethnic minorities and poorer individuals during the pandemic [3]. Food insecurity is associated with negative health consequences in both children and adults, including lower nutrient intake [4,5,6], physical and mental health problems [7,8,9,10], and higher levels of chronic disease [11,12].

While food security is a measure of the ability to obtain food, the main cause of food insecurity is low income [13]. According to the federal report, Household Food Security in the United States in 2019, 34.9% of households with annual incomes below the poverty line experienced food insecurity compared to 5.1% of those with incomes above 185% of the poverty line [14]. In 2018, the poverty rate was 11.8%, and 38.1 million people lived in poverty, although researchers argue that the current poverty measures underestimate the actual number [15]. Like food insecurity, poverty disproportionately affects African Americans (20.8%), Hispanics (17.6%), single family households with children and female (39.1%) and male (18.7%) householders [16]. Areas with higher poverty rates are also more likely to be a food desert, an area characterized by poor access to healthy and affordable food [17,18]. Food deserts are more prevalent in primarily Black and Hispanic neighborhoods [17]. Transportation can be limited in these areas, and convenience stores that are closer than full-service supermarkets sell food at higher prices [19]. A variety of solutions to limited food access have been proposed and tested, including farmers markets, incentives for store owners who make healthy food more accessible, marketing strategies, and online grocery shopping [20]. It is likely that a combination of these creative approaches and others will be needed to improve food access and food security in the United States.

One potential approach involves the use of meal kits. Meal kits are boxes or bags of fresh and shelf-stable ingredients for one or more meals, along with a step-by-step recipe showing how to cook each meal at home [21]. Meal kits are a convenient meal planning and preparation strategy for busy families and have increased in popularity in recent years. Growing from $1.6 billion in 2016 to $4.65 billion in 2017, meal kit services may be one way to overcome the barriers related to food access and eating healthy [22]. Meal kits are not only growing in popularity through delivery service models but also within in-store retail outlets [23]. Most meal kit subscribers are individuals with higher income [21], though lower-cost meal kit programs exist such as Dinnerly and EveryPlate.

Few studies have assessed the usability and acceptability of meal kit services, especially among consumers with low income. Utter et al. completed two studies in New Zealand assessing the acceptability of meal kit interventions among families with adolescents. Ten families were recruited from a school with ethnic and socioeconomic diversity and provided with five dinners per week for eight weeks in the first study [24], while nine families were recruited from a youth health clinic and provided with five meals per week for four weeks in the second study [25]. Intervention utilization and acceptability were high in both studies and positive aspects of the studies included the quality of ingredients, ability to try new foods, easy-to-follow recipes, ease of preparation, adolescent involvement and enjoyment in cooking, experience of eating together, and perceived positive impact on nutrition [24,25]. Frequency of family meals and food security status were assessed in the second study and increased significantly after the meal kit program [25]. While both of Utter’s studies demonstrated the feasibility of meal kit interventions and positive impact of increasing family meal frequency, comprehensive data are lacking on the effect of meal kit interventions on diet quality, physical health parameters, and family mealtime interactions, as well as the affordability of meal kit services that consumers purchase.

Another meal kit study was conducted in a pediatric weight management clinic to assess the acceptability and feasibility of a meal kit intervention [26]. In this pilot study, patients and their families (n = 15) were provided meal kits with non-perishable foods and a gift card to purchase perishable ingredients. After participating in the intervention, adolescent participants (n = 4) and caregivers (n = 8) completed focus groups to determine barriers to cooking at home and participants’ impressions of the meal kits. Participants found the meal kit intervention to be acceptable and found the pediatric weight management clinic to be an acceptable setting to offer the program. Participants listed several barriers to home cooking, including the cost of healthy food, time to prepare meals, lack of food preparation and meal planning knowledge, and picky eaters. Participants discussed how the intervention addressed all of the barriers, but the authors did not report any discussion around the cost of healthy food. Like the studies referenced previously, this study demonstrated the feasibility and acceptability of a small-scale meal kit program.

Popular consumer meal kits range in price from USD $5 to $11 per serving [27,28]. It is unclear if this is an appropriate or realistic price point for families with low income. If meal kits are acceptable for families with low income, it is important to identify an appropriate willingness to pay (WTP), which has not yet been assessed in nutrition-focused meal kit studies. Therefore, the purpose of this pilot study was to determine the utilization, acceptability, and WTP of a culturally acceptable healthy meal kit intervention among African American families with low income.

## 2. Materials and Methods

### 2.1. Slice and Spice Meal Kit Intervention

A meal kit program, Slice and Spice, was developed for this intervention. In an effort to keep costs low and serve families with low income in the local community, researchers partnered with a public high school’s Institute of Culinary Arts, a Career and Technical Education (CTE) program, to create the meal kit intervention. Since labor and delivery costs contribute significantly to the overall costs of a commercial meal kit, partnering with the culinary arts program for the procurement of ingredients and kit preparation allowed the researchers to decrease these costs for the intervention. In addition, this high school is located in a community with higher rates of poverty, making it more accessible for families experiencing food insecurity to pick up their meal kit.

Participants received meal kits weekly for six weeks. Each meal kit contained three recipes that served four people. Recipes and other aspects of the meal kit program (e.g., location to pick up meal kit, preference for fresh or shelf-stable food, and willingness to try new foods) were selected based on input from focus groups with the target audience (unpublished data). All recipes met the following nutrition criteria per serving, which were selected based on the 2015–2020 Dietary Guidelines for Americans recommendations: less than 800 kilocalories; <1/3 percent daily value for sodium, saturated fat, and added sugars; 0 g trans fat, >10 percent daily value for either vitamin A, vitamin C, vitamin D, calcium, iron, potassium, or fiber; at least one cup vegetables; at least two ounces of grains (emphasizing whole grains); and at least two ounces of protein foods [29]. Participants received all ingredients to cook the meals (some of which were already processed such as chopped onions and peeled garlic), recipe cards, a cooking incentive (e.g., meat thermometer, cutting boards, measuring cups and spoons), and a nutrition handout each week. Nutrition handouts were created by this study’s research coordinators and reviewed by the Principal Investigator, all of whom are Registered Dietitian Nutritionists with graduate degrees in nutrition. The recipe cards and nutrition handouts were designed by a graphic designer, printed on 4” × 8.5” durable paper that could easily be cleaned if food spills occurred during cooking, hole punched, and bound with a metal ring to create a recipe and nutrition handout collection during the intervention. All intervention components were provided to participants free of charge. All study procedures were approved by the University of Florida Institutional Review Board (IRB; #201802455), and participants provided signed consent.

### 2.2. Participants

Participants were recruited from various locations in the study catchment area. Recruitment sites included the nearby public schools (targeting support staff and parents), a medical office for children with special health care needs, a food bank, a church, and a community resource center. This study was completed in two waves, and many participants from wave one informally recruited participants for wave two through word-of-mouth methods. Participants were recruited and screened for eligibility in person or over the phone using an approved script.

Individuals were eligible to participate in this study if they met the following criteria: identify as African American, 18 years or older, have a child younger than 18 years of age living in the household, not living with someone who had a condition that restricted food intake/had severe food allergy, identified as the main food preparer, and met low-income qualifications. Specifically, individuals must qualify for SNAP, Medicaid, Temporary Assistance for Needy Families, Special Supplemental Nutrition Program for Women, Infants, and Children, or have a monthly gross income less than or equal to 185% of the US Poverty Income Guidelines [30].

Sample sizes were estimated based on an anticipated change in dietary quality from baseline measures to post-intervention and long-term follow up using R version 4.0.5. The required sample size of 40, 30 or more remaining at 6 months, was generated based on the assumption that the minimum observed effect size would be 0.6 or greater, with an alpha of 5%, power of 80%, attrition rate of 25%, and intra correlation between the same subject of 0.5.

### 2.3. Data Collection

A one-group, pre–post-test design using a double post-test was implemented to assess the immediate and long-term effects of the meal kit program. While three assessment time points were used, only results from baseline and immediately post-intervention will be reported in this manuscript because they are most relevant to the focus of this paper. Participants attended a baseline data collection event where they were provided with dinner while completing all required baseline assessments. Participants were provided their first meal kit at the end of the baseline dinner. Each week for the next five weeks participants picked up their meal kit and completed a short survey measuring satisfaction and utilization of the meal kits from the previous week. Participants returned for another dinner data collection event after the six-week intervention to complete post-intervention data collection, which followed the same format as the baseline data collection. Finally, participants returned for one final dinner data collection event to complete the second post-test at long-term follow up (LTFU) approximately six months after the intervention concluded. Baseline, post-intervention, and LTFU assessments each lasted approximately 90 min. Participants received $50 in cash compensation for completing each of the three data collection events ($150 total).

### 2.4. Outcome Measures

#### 2.4.1. Sociodemographic Characteristics

A demographic survey was created by the research team to collect participants’ sociodemographic characteristics. Height and weight measurements were obtained at baseline, post-intervention, and LTFU by trained research staff to calculate BMI. Standard procedures were used to measure height with a stadiometer (to the nearest 0.1 cm) and weight using a digital scale (to the nearest 0.1 lb) [31]. Weight in lb was divided by 2.2 to convert weight into kilograms (kg). BMI was calculated as weight (kg) divided by height (meters) squared.

#### 2.4.2. Food Security Survey

Food security status was measured at baseline and LTFU with the 18-Item US Household Food Security Survey Module [32], a reliable and validated tool [33]. Food security status was calculated in accordance with the United States Department of Agriculture (USDA) Guide to Measuring Household Food Security, and raw scores were categorized into four food security categories: high food security (raw score 0), marginal food security (raw scores 1–2), low food security (raw scores 3–7), and very low food security (raw scores 8–18) [32]. Participants were also classified in two food security categories, food secure (raw scores 0–2) and food insecure (raw scores 3–18).

#### 2.4.3. Knowledge and Use Survey

Participants completed a survey at baseline that assessed their knowledge and use of meal kit services. A meal kit was described as “a box of healthy, perishable and nonperishable ingredients for one or more meals, along with a step-by-step recipe describing how to cook each meal at home”. Questions included topics such as participant’s familiarity with meal kits, if they considered buying a meal kit, if they have bought a meal kit previously, preference for receiving a meal kit (e.g., delivery or pick up), if certain situations would encourage or discourage participants from using meal kits, frequency of food shopping at various retail outlets, the importance of several factors when making food purchasing decisions, and sources of information on foods to eat and avoid. Several questions were adapted from the International Food Information Council Foundation’s Food and Health Survey [34].

Willingness to pay (WTP) in USD was also assessed in the Knowledge and Use Survey. WTP is the “maximum price a given consumer accepts to pay for a product or service” [35], and is one method to determine an acceptable cost of a good. WTP was assessed by asking, “How much would you be willing to pay per meal (for one person) included in the meal kit?”, “How much would you be willing to pay for a meal kit with ingredients for one recipe to feed four people?”, and “Would you be willing to pay (3x number above) for a meal kit with ingredients for three recipes, each to feed four people? If not, how much would you be willing to pay for a meal kit with ingredients for three recipes, each to feed four people?”

#### 2.4.4. Acceptability Survey

Questions from the Knowledge and Use Survey were rephrased to assess the acceptability of the intervention in the Acceptability Survey, which was administered at the post-intervention time point. Additional questions covered topics such as participants’ willingness to use SNAP benefits on meal kits, level of satisfaction with the meal kits overall and the amount of preparation required, impact of meal kits on other food purchasing decisions, and which recipes in the meal kits participants would make again. Research staff asked participants a subset of open-ended questions about the meal kits and wrote detailed notes of their responses. Questions included what participants liked most and least about the meal kit service; what changes should be made to improve the meal kit service; if participants made any of the recipes again and if they made any changes to the recipes; general thoughts about the quality of ingredients, recipe cards, nutrition handouts, and cooking incentives; barriers faced with the project and strategies to overcome the barriers; if they would participate in the project again; and best ways to recruit families to participate in the meal kit service.

#### 2.4.5. Weekly Process Survey

Participants completed weekly process surveys during the intervention that included questions pertaining to the meal kit they received each week. Participants were asked if they prepared each of the three recipes, if any changes were made to the recipes, how much they liked the recipes, their WTP for each individual meal, their WTP for the three meals included in the meal kit, if they used the cooking incentive they received, and if they used the information in the nutrition handout while cooking or eating.

### 2.5. Data Analysis

The Demographic Survey, Food Security Survey, Knowledge and Use Survey, Acceptability Survey, and Weekly Process Survey were analyzed using descriptive statistics, including mean ± standard deviation for continuous variables and n (percentage) for categorical variables. Short answer, qualitative responses to questions related to participants’ likes and dislikes about the program were summarized using an inductive approach. Representative quotes were selected to create a dialogue that illustrates major findings [36]. Statistical analyses were conducted in International Business Machines Corporation (IBM) Statistical Package for the Social Sciences (SPSS) Statistics for Macintosh, version 26 (Aramonk, NY, USA).

A cost analysis of the meal kits was conducted. Ingredient costs were estimated using invoices from orders placed by the culinary program’s chef from a large food distributor, a local produce company, and a supermarket. Packaging costs and other implementation costs were also estimated. The results of the cost analysis were compared against participants’ WTP for meal kits in this study to better understand if participants were willing to pay a price for the meal kits that covered food and packaging costs.

## 3. Results

### 3.1. Sociodemographic and Health Characteristics

Participant baseline demographic and health characteristics are listed in Table 1. To summarize, participants were primarily middle aged (42.5 ± 13.8 years), female (88.9%), and experienced food insecurity (66.7%). Participants had household incomes ranging from <$15,000 to averages within the range $50,000–$74,999, and most participants (93.1%) had a household income of <$50,000 per year. Most participants (72.2%) reported that their highest level of education was high school or some college. The average household included 1.8 ± 1.0 adults and 2.3 ± 1.2 children. The majority (66.7%) of participants had obesity, and mean BMI indicated class II obesity [37].

### 3.2. Participant Experience with Meal Kits

While 60% of participants had heard of a meal kit at the start of this study, only 14.3% had actually bought a meal kit (Table 2). After the intervention, 86.1% of participants thought about buying a meal kit, and 16.7% (n = 6) had bought a meal kit since this study began (five of whom did not answer this question affirmatively at baseline). While participants picked up their meal kits from a central location in the community, over half (52.9%) would prefer to have a meal kit delivered to their home. Over half (58.3%) of participants who were eligible for SNAP benefits would “definitely” be willing to use their SNAP benefits to purchase meal kits if possible.

### 3.3. Meal Kit Utilization and Acceptability

Participants prepared most of the meal kit recipes. The preparation rates by recipe ranged from 80.6% to 97.2%. Vegetarian recipes were prepared least often (all 80.6%). Of the 18 recipes that were offered as part of the intervention, participants prepared on average 16.4 ± 2.0 of the recipes, indicating a high utilization rate. Nearly half (44.4%) of participants prepared all 18 recipes. Participants reported modifying the recipes frequently, with approximately one-quarter to one-half of participants modifying each of the recipes.

Participants received a cooking incentive and a nutrition handout each week and were asked about their use of these materials the following week. Most participants reported using the cooking incentives, with usage rates ranging from 71.4% to 88.9%. Lastly, participants were asked about their use of the nutrition information on the nutrition handouts. Use of the information was high and ranged from 65.7% (information about nutrition facts labels) to 100% (information about healthier seasonings).

In addition to participants’ utilization of the meal kits, they were surveyed on the acceptability of the meals (Table S1). The recipe with the lowest acceptability (Spaghetti Squash with Meat Sauce) was still acceptable to over two-thirds of participants. Greater than 90% of participants indicated a high level of acceptability (classified as liking recipes “a great deal” or “somewhat”) for dishes with the following protein sources: 1 out of 1 pork recipe, 5 out of 6 seafood recipes, 3 out of 4 poultry recipes, 1 out of 3 beef recipes, and 0 out of 4 vegetarian (bean) recipes.

### 3.4. WTP for Meal Kits

General WTP for meal kits was assessed at baseline and post-intervention (Table 3). At baseline, participants stated an average WTP of $30.20 ± 15.26 for one meal with four portions and $74.03 ± 51.02 for three meals with four portions. WTP for meal kits increased when reassessed post-intervention to $32.96 ± 15.94 for one meal with four portions and $88.61 ± 47.47 for three meals with four portions.

Participants were also asked each week about their WTP for the individual meals and the meal kit (all three meals) provided as part of the intervention (Table 4). Individual meal (with four portions) WTP ranged from to $14.82 ± 8.59 (Spaghetti Squash with Meat Sauce) to $27.37 ± 16.31 (Summer Salmon). WTP for the meal kit with three meals ranged from $48.44 ± 38.14 (week 3 meals) to $61.77 ± 48.38 (week 4 meals). All weekly assessments of meal kit WTP were lower than the meal kit WTP assessed at baseline and post-intervention.

### 3.5. Cost Analysis Results

Food and packaging costs were estimated for each recipe and are listed in Table 4. Individual recipe costs were calculated by determining the price of each portion of each ingredient for each recipe. In addition to the ingredients, each meal (with four portions) cost an additional $8.52 to produce, which covers the cost of the following items divided equally over all meals produced for this study: bottles of olive oil, salt & pepper shakers, ice packs, insulated thermal bags, reusable tote bags, individual ingredient packaging, ingredient sticker labels, recipe cards, and nutrition handouts. The number is an estimate of the total food/packaging cost and does not take the following variable and fixed costs into account: labor hours to produce meal kits, cost of facilities where meal kits were produced, electricity, cooking incentives given to participants each week, and stipend provided to the culinary program for assisting with this study, or markup for profit.

Food and packaging costs per recipe ranged from $12.17 (Bean and Rice Burrito) to $23.48 (Summer Salmon) depending on the cost of ingredients in each recipe, and the average food/packaging cost per recipe was $16.88. Average WTP was higher than food/packaging cost for 14 out of the 18 recipes. The total percentage of participants who stated a WTP equal to or higher than the food/packaging cost is listed in Table 4. This percentage varied by recipe, with the lowest percentage (32.4%) willing to pay for the Spaghetti Squash with Meat Sauce and Tuna Pasta Casserole and highest percentage (77.1%) willing to pay for the Baked Pork Chops with Vegetables. Finally, some participants were unwilling to pay for a meal kit and stated a WTP of $0.00. The percentage of participants unwilling to pay for meal kits is noted in Table 4 under the heading “% Unwilling to Pay”.

Food/packaging costs were also compared to the WTP amounts that participants reported at the post-intervention data collection time point. At post-intervention, participants were willing to pay $32.96 ± 15.94 for one meal with four servings, which is higher than the food/packaging cost for all of the individual recipes. Participants also reported a WTP of $88.61 ± 47.47 for the meal kit (three meals, each with four servings). This amount is higher than the weekly food/packaging costs for the meal kits. Therefore, food/packaging cost per recipe was similar to average WTP when assessed weekly, but always lower than average WTP when assessed at the post-intervention time point.

### 3.6. Meal Kit Acceptability and Effect on Food Purchasing Decisions at Post-Intervention Time Point

Overall, participants were satisfied with the meal kit program when assessed post-intervention. Three-quarters of participants (75.0%) reported being “very satisfied” with the meals, seven (19.4%) were “somewhat satisfied”, and two (5.6%) were “neither satisfied nor dissatisfied”. All but one (97.2%) participant felt that there was “just the right amount of preparation required” in the meal kits, and the other participant felt the meal kits involved too much food preparation. Many participants felt that the meal kits influenced their other food purchasing decisions (Table 5). Over half (58.3%) of the participants reported shopping at the grocery store less, while eight (22.2%) reported shopping at the grocery store more. Meal kit dinners may have replaced meals eaten at restaurants, with one-quarter (25.0%) of participants reporting buying food from fast food restaurants less and one-quarter (25.0%) reporting buying food from other restaurants less.

### 3.7. Participant Qualitative Feedback on Meal Kit Intervention

Participants were asked a series of open-ended questions about the meal kits after they completed the intervention. When asked what they liked about the program, participants mentioned that “it was very convenient” because “everything was already measured in there” and included “thorough directions”. Participants also appreciated how the meal kits helped them save time because they “didn’t have to go grocery shopping” and the meal kits had “all the stuff in it you need.” Participants appreciated that they had the opportunity to try “something new with different recipes”. Another benefit was “what it did for the family”, including allowing “bonding time with family” and “helping families have healthy, simple meals”. Participants found cooking meal kits to be a “fun experience” that helped them “be more conscious about what [they’re] eating”. It also helped them recognize “the overall value of ingredients” by stating, “You would pay $8.99 for Chinese takeout and it doesn’t taste as good. It made me realize the importance of good ingredients”.

That being said, participants also discussed aspects of the program that they did not like and ways to improve it for future interventions. Every meal kit contained four portions, and participants mentioned that “it didn’t feed everyone”. Some participants found that “pick up was difficult” because they “lived a little far away” or because “the time of day to pick up the meals” did not fit their schedule. Participants received a fixed menu with the same 18 recipes, but participants “didn’t like some of the recipes”, “not being able to choose the recipes”, and preferred a “menu of meals to choose from”. Specific recipe improvements that were suggested included adding “more fruit”, “providing a healthy drink with the kit”, and adding “extra seasoning to the meal kits” because some of the meals were “bland”. Participants also discussed how they modified recipes each week, which included adding beef to vegetarian recipes, “adding seasoning and some cheese for taste”, and omitting beans such as chickpeas and black beans for “dietary influences”. Adding seasonings, sauces, and/or cheese were the most common modifications. If participants had fewer than four people in the house, they mentioned eating the remaining portions as leftovers. Families larger than four members would either prepare “some other meal”, “[tell] children to have a small portion”, or purchase similar ingredients to “double to feed the family of eight”.

Participants offered their insight on the recipe cards, nutrition handouts, and cooking incentives. Participants provided overwhelmingly positive feedback on the recipe cards, and especially appreciated the “good quality” and “sturdiness”, how they were “easy to read” and “very detailed so children could help out” and “one foster child set ingredients out and the other read ingredients list”. Participants also appreciated the inclusion of “nutrition facts labels”, which “helped keep counts every day”. Participant interest and use of the nutrition handouts varied, from not using them because they “felt they already knew it”, “read[ing] some of them”, to being the “first thing we look at” and using them to make “a game within the family to try and guess which recipe was the healthiest”. Cooking incentives that were most helpful and “came in very handy” included the cutting boards because “it makes such a difference to have colored ones for each type of food” and the meat thermometer, which several participants mentioned they “didn’t already have”. Even if participants already had the incentives, they were able to “replace some older tools” with the new items. Participants suggested including a vegetable peeler, can opener, knife sharpener, and kids knives in future interventions.

## 4. Discussion

The aim of this pilot study was to determine the utilization, acceptability, and WTP for a healthy meal kit intervention for families with low income. Findings revealed that participants prepared and liked healthy meals offered in the intervention, despite few trying meal kits before participating in this study. Meal preparation rates were high throughout the intervention, with individual recipe preparation rates ranging from 80.6% to 97.2% and almost half of the participants preparing all 18 recipes. Throughout the intervention, several participants missed a weekly meal kit pickup and could not be reached to schedule an alternative pickup time, so participants were unable to make those meals. The average utilization of 16 out of the 18 recipes takes these missing pickups into account, so the average utilization rate of participants who picked up the meal kits was even higher. Most recipes were rated with a high level of acceptability, and participants frequently used the included cooking incentives to prepare their meals. Another important part of this study was comparing participants’ WTP for the meal kits to the food/packaging costs. This comparison will help researchers begin to understand the affordability of this meal kit model for the desired target audience. During the intervention, participants reported an average WTP that was similar to the food/packaging cost. At the post-intervention time point, the average WTP was higher than the food/packaging cost for all recipes.

This study adds to the literature by determining WTP for meal kits among participants with low income. Participants stated a WTP for a meal kit (3 meals with 4 servings each) of $74.03 ± 51.02 at baseline, between $48.44 ± 38.14 and $61.77 ± 48.38 during the intervention, and $88.61 ± 47.47 at the end of the intervention. The least expensive national commercial meal kit service in the United States, Dinnerly, costs $68.87 for the same number of meals, while the two most popular services, Blue Apron and Hello Fresh, cost more at $95.88 and $109.87, respectively [27,28,38]. Based on the WTP data collected in the present study, most commercial meal kit services, which are primarily utilized by individuals with higher income [21], are priced too high for consumers with lower income. If meal kit companies wish to increase sales among customers with lower income, they will need to create a product at a price point that is acceptable or adjust prices of their current meal kits accordingly. The fact that more participants thought about purchasing a meal kit and purchased a meal kit after the intervention suggests that families with low income may be a new audience to target for affordable meal kits. Increasing awareness of meal kit services is also needed for this demographic group, as only 60% of participants had heard of meal kits before, compared to 73% of consumers surveyed from a representative sample of the population in 2017 (which has likely increased since then since meal kits have grown in popularity) [21]. Some meal kit companies sell meal kits at grocery stores, which may be a helpful way of reaching consumers with lower income (meal kits that are not delivered are more affordable), and SNAP-eligible individuals could purchase the meal kits with their SNAP benefits [39]. Although the majority of participants in this study preferred to receive a meal kit through delivery to their homes, 35.3% preferred to pick them up from a central location such as a grocery store. Or the USDA may consider extending SNAP benefits to be used on meal kit delivery, as they have recently expanded SNAP for online grocery shopping. The 2014 Farm Bill approved piloting use of SNAP benefits through online grocery stores, and the pilot is currently offered in 47 states across the country [40]. SNAP beneficiaries can shop for and purchase eligible foods online, though they cannot use their SNAP benefits for delivery or service charges [27]. A preliminary study on online grocery shopping with SNAP benefits reported a low participation rate [41], so it may be worthwhile for the government to consider alternative channels for electronic use of SNAP benefits, such as meal kit delivery services, and find ways to remove delivery fees to increase participation rates.

It is also worthwhile to consider alternative models for procuring and preparing meal kits for families with low income if commercial meal kit companies are unable to create a product at an appropriate price point and/or if SNAP cannot be used for meal kit delivery. One potential model involves partnering with high school Career and Technical Education (CTE) programs that are located in communities with low income, as was done in this study with the high school’s Institute of Culinary Arts. Such a project not only provides an opportunity for the students to serve their community but is aligned with their CTE standards and benchmarks (e.g., food safety guidelines, basic principles of nutrition, and resource management) and can provide a true service-learning experience [42]. While the meal kit program partnership was successful with the CTE program for this study, it is unclear how replicable this model will be in other public school districts, therefore, interviews with the culinary program chef and school district administrators should be included in future studies to more accurately discover all costs to complete this study and to better understand how realistic this model is from stakeholders within a public school system.

The findings of this study corroborate published research on meal kits, which show that healthy meal kit interventions can be logistically feasible, highly utilized, and acceptable to participants [24,25,26,43]. In the present study, meal kit utilization ranged from 80.6% to 97.2%. One published pilot study with 10 families evaluated intervention utilization and reported that participants prepared 85% of the meal kit meals. Meals were delivered to participants and offered through meal kit businesses [24]. Utilization was lower in another study that required participants to order shelf-stable food boxes online and purchase fresh produce with produce checks [44,45]. On average, 65% of households ordered a food box, and 65% of households that received produce checks redeemed them even though they were offered free of charge, could be ordered through the internet or telephone, and delivered to participants’ houses [45]. Differences between this study and the present study with a higher utilization rate include a longer intervention period (25 months versus 6 weeks), different delivery method (mail versus pickup at central location), and types of food (shelf-stable ingredients with produce checks versus fully composed meals with all ingredients and recipes) [45]. Utilization was not reported in other meal kit studies [25,26,43]. Previous studies have also inquired about characteristics of the meal kit interventions that participants enjoyed. Some positive attributes included the variety and quality of ingredients [24], ease of preparation [24], helpfulness in meal planning and grocery shopping [24,26], helpfulness in identifying portion sizes [26], and sharing cooking responsibilities with other family members [43]. Participants in the current study rated the intervention with an overall high level of acceptability. Like participants in the previous studies, participants in this study mentioned how they enjoyed the variety and quality of ingredients, convenience and ease of preparation, and helpfulness with grocery shopping because they did not need to shop as often. Participants in a previous study cited challenges with preparing the meal kits, including not having enough space in the kitchen, creating too many dirty dishes, and taking too much time to prepare the meals [24]. These sentiments were not expressed by participants in this study, though they did cite some challenges around picking up the meal kits, needing to modify recipes by omitting ingredients or adding seasonings, and scaling recipes up to feed larger families. None of the published meal kit studies reported using a similar model of a community-based meal kit program implemented through a high school, nor did they report on the cost of the program or WTP among participants.

This study was conducted before the COVID-19 pandemic, but it is important to consider the implications of this study within the context of our current economic and health landscape. Food insecurity was projected to impact 54 million Americans after the onset of the COVID-19 pandemic, which is an increase of 17 million Americans compared to 2018 food insecurity rates [46]. The federal government has strengthened nutrition assistance programs since the onset of the pandemic [47], but these efforts are not robust enough as food banks report serving more individuals, 40% of whom are visiting the food banks for the first time [48]. Innovative programs such as this one may help improve food security and food access if more widely available to those facing food insecurity.

This study has several strengths and limitations. Participant utilization and acceptability were assessed with a variety of measures, which were key to determining the appropriateness and feasibility of the intervention. Unlike previous meal kit research, participants were asked about their WTP for the meal kits to better understand if meal kits are affordable for families with low income. The intervention was conducted in a population with low income, who do not typically purchase meal kits but may benefit from healthy meal kits because they help reduce barriers to accessing, cooking, and eating healthier meals. Another strength was including questions about willingness to use SNAP benefits on meal kits, which is important for both commercial meal kit companies for advertising purposes and the USDA, who may consider extending SNAP purchases to meal kit delivery. Finally, a novel model was tested that included a high school culinary program. This was mutually beneficial to the researchers who did not need to coordinate meal kit preparation and to the students who were able to practice food preparation techniques and be actively involved in the research process. Future studies should include the students as study subjects to get a better understanding of how participating in this project affected their dietary behaviors, career aspirations, motivation for learning, critical thinking, and community engagement. Limitations include the small sample size (n = 36), shorter intervention time frame (6 weeks), and lack of a control group. Due to initial difficulties with participant recruitment, the researchers forwent a control group and conducted the pilot study as described. Additionally, data were only collected from the main food preparer in the family, though other family members (children, spouses, etc.) and high school students involved in developing the kits may have been impacted by the meal kits. Finally, while the WTP and cost analysis results allowed researchers to begin to understand if the meal kit program may be affordable for the target audience, the results should not be overstated. Definitive claims cannot be made about the affordability of meal kits for families with low income solely from this study because of the small sample size of this pilot study, large variability in participants’ WTP for the meal kits, as well as exclusion of labor and facility costs due to the partnership with the CTE program. Additionally, WTP estimates collected using open-ended contingent valuation questions (as used in this study) are subject to hypothetical bias.

## 5. Conclusions

In conclusion, the results of this study suggest that the meal kit program model tested in this study was possible to implement, highly utilized, and acceptable to participants. Participants reported a WTP that suggests the program may be affordable to families with low income if implemented in a way that minimizes facilities, utilities, and labor costs. The results of this study add to the meal kit literature by testing a novel meal kit program model, assessing the utilization and acceptability of meal kits with African American families with low income, and collecting information on participants’ WTP.

## Figures and Tables

**Table 1:** Participant Sociodemographic and Health Characteristics.

Characteristic	Total (*n* = 36)
Age in years, mean ± standard deviation	42.5 ± 13.8
Gender, *n* (%)	
Male	4 (11.1)
Female	32 (88.9)
Food security status, *n* (%)	
High food security	4 (11.1)
Marginal food security	8 (22.2)
Low food security	9 (25.0)
Very low food security	15 (41.7)
Income ^a^ (*n* = 29), *n* (%)	
<$15,000	6 (20.7)
$15,000–$24,999	8 (27.6)
$25,000–$34,999	6 (20.7)
$35,000–$49,999	6 (20.7)
$50,000–$74,999	3 (10.3)
Education, *n* (%)	
Less than high school	2 (5.6)
High school graduate or GED	9 (25.0)
Some college	17 (47.2)
Associate’s degree/technical school graduate	2 (5.6)
Baccalaureate degree	4 (11.1)
Advanced college degree	1 (2.8)
Other	1 (2.8)
Number of adults in household, mean ± standard deviation	1.8 ± 1.0
Number of children in household, mean ± standard deviation	2.3 ± 1.2
BMI category, *n* (%)	
Underweight	0 (0.0)
Normal weight	5 (13.9)
Overweight	7 (19.4)
Obesity	24 (66.7)
BMI (kg/m^2^), mean ± standard deviation	35.4 ± 9.2
Body Fat %, mean ± standard deviation	38.6 ± 11.3

^a^ Household income was retrospectively assessed at LTFU, which limited responses to *n* = 29.

**Table 2:** Participant Experience with and Preferences for Meal Kits.

Experience	Amount at Baseline, *n* = 35 % (*n*)	Amount at Post-Intervention, *n* = 36 % (*n*)
Heard of a meal kit	60.0 (21)	
Thought about buying a meal kit	42.9 (15)	86.1 (31)
Bought a meal kit	14.3 (5)	16.7 (6)
Preference for receiving a meal kit		
Delivered to home		52.9 (18)
Picked up at central location (e.g., church, school, community center)		35.3 (12)
Picked up at grocery store		5.9 (2)
Other		5.9 (2)
Willingness to use SNAP benefits to purchase meal kit		
Definitely		58.3 (21)
Probably		0.0 (0)
Possibly		19.4 (7)
Probably not		0.0 (0)
Definitely not		0.0 (0)
Do not receive SNAP benefits		22.2 (8)

**Table 3:** Participant WTP for Meal Kits in General.

Item	WTP at Baseline ($) (Mean ± Standard Deviation)	WTP at Post-Intervention ($) (Mean ± Standard Deviation)
WTP for one meal with one portion	11.61 ± 6.13	12.22 ± 5.79
WTP for one meal with four portions	30.20 ± 15.26	32.96 ± 15.94
WTP for three meals, each with four portions	74.03 ± 51.02	88.61 ± 47.47

**Table 4:** Participant WTP and Costs per Recipe.

Recipe	WTP ($) (Mean ± Standard Deviation)	Food and Packaging Cost per Recipe ($)	% Willing to Pay ≥ Food and Packaging Cost (%)	% Unwilling to Pay (%)
Summer Salmon	27.37 ± 16.31	23.48	47.1	0.0
BBQ Chicken Pizza with Side Salad	19.06 ± 15.75	15.80	38.9	0.0
Pasta with Chickpeas, Tomatoes, and Spinach	15.71 ± 12.23	12.47	54.5	15.2
All Recipes in Week 1 Meal Kit	53.49 ± 34.90	51.75	38.9	0.0
Vegetable and Shrimp Stir Fry	21.91 ± 11.76	22.22	44.1	0.0
Chicken Salad with Orange Vinaigrette	16.04 ± 6.44	19.49	37.1	0.0
Spaghetti Squash with Meat Sauce	14.82 ± 8.59	17.61	32.4	8.8
All Recipes in Week 2 Meal Kit	51.37 ± 28.38	59.32	42.9	0.0
Tuna Pasta Casserole	16.11 ± 11.31	16.24	32.4	5.9
Black Bean Quesadilla with Corn, Tomato, and Avocado Salad	16.72 ± 13.94	14.20	56.2	6.3
Stuffed Bell Pepper Soup	19.35 ± 26.33	15.67	37.5	6.3
All Recipes in Week 3 Meal Kit	48.44 ± 38.14	46.11	39.4	0.0
Ginger Glazed Mahi Mahi	26.81 ± 22.53	20.04	45.7	2.9
Baked Pork Chops with Vegetables	25.26 ± 23.39	14.81	77.1	0.0
Hearty Spinach Salad	19.28 ± 16.19	18.21	42.9	2.9
All Recipes in Week 4 Meal Kit	61.17 ± 42.37	53.06	48.6	0.0
Shrimp Scampi Bake	24.43 ± 14.48	21.39	42.9	0.0
Bean and Rice Burrito	15.11 ± 9.47	12.17	54.3	5.7
Barley Jambalaya	19.79 ± 13.37	15.27	48.6	2.9
All Recipes in Week 5 Meal Kit	59.24 ± 56.07	48.83	51.4	0.0
Tilapia Creole	23.80 ± 20.18	15.95	43.7	0.0
Vegetable Stroganoff	17.15 ± 14.31	14.17	56.2	12.5
Stir Fry Vegetables and Beef	22.32 ± 17.37	14.71	70.6	0.0
All Recipes in Week 6 Meal Kit	61.77 ± 48.38	44.83	58.8	0.0

**Table 5:** Ways Meal Kits Impacted Other Food Purchasing Decisions.

Indicator	Amount % (n) ^a^
Shopped at the grocery store less	58.3 (21)
Bought food at fast food restaurants less	25.0 (9)
Shopped at the grocery store more	22.2 (8)
Bought food at other restaurants less	25.0 (9)
Bought food at fast food restaurants more	2.8 (1)
Bought food at other restaurants more	0.0 (0)

^a^ Select all that apply response option.

## Data Availability

The data in this study are available upon request by the corresponding author.

## Supplementary Materials

The following are available online at https://www.mdpi.com/article/10.3390/nu13082881/s1, Table S1: Recipe Acceptability Assessed during the Intervention.
