# Supplementary material for: Acceptability and Willingness to Pay for a Meal Kit Program for African American Families with Low Income: A Pilot Study
Source: Nutrients. 2021 Aug 21;13(8):2881. doi: 10.3390/nu13082881 (PMC8398692; doi:10.3390/nu13082881)
Supplement: Supplementary file 1 [file nutrients-13-02881-s001.zip › nutrients-1329529-supplementary.pdf]

Table S1. Recipe Acceptability Assessed During the Intervention

Recipe	Recipe Acceptability n (%)				
	**Liked a Great Deal**	**Liked Somewhat**	**Neither Liked nor Disliked**	**Disliked Somewhat**	**Disliked a Great Deal**
Summer Salmon	31 (88.6)	4 (11.4)	0 (0.0)	0 (0.0)	0 (0.0)
Shrimp Scampi Bake	31 (88.6)	4 (11.4)	0 (0.0)	0 (0.0)	0 (0.0)
Pork Chops with Roasted Vegetables	33 (94.3)	2 (5.7)	0 (0.0)	0 (0.0)	0 (0.0)
Vegetable and Shrimp Stir Fry	27 (79.4)	7 (20.6)	0 (0.0)	0 (0.0)	0 (0.0)
BBQ Chicken Pizza with side salad	23 (65.7)	10 (28.6)	2 (5.7)	0 (0.0)	0 (0.0)
Tuna Pasta Casserole	20 (58.8)	12 (35.3)	1 (2.9)	0 (0.0)	1 (2.9)
Chicken Salad with Orange Vinaigrette	22 (64.7)	9 (26.5)	2 (5.9)	1 (2.9)	0 (0.0)
Hearty Spinach Salad	28 (84.8)	4 (12.1)	0 (0.0)	1 (3.0)	0 (0.0)
Stir Fry Vegetables with Beef	28 (82.4)	3 (8.8)	3 (8.8)	0 (0.0)	0 (0.0)
Tilapia Creole	21 (70.0)	6 (20.0)	1 (3.3)	2 (6.7)	0 (0.0)
Barley Jambalaya	17 (50.0)	12 (35.3)	1 (2.9)	3 (8.8)	1 (2.9)
Black Bean Quesadillas with Corn, Tomato, and Avocado Salad	16 (55.2)	9 (31.0)	0 (0.0)	3 (10.3)	1 (3.4)
Stuffed Pepper Soup	13 (44.8)	12 (41.4)	1 (3.4)	2 (6.9)	1 (3.4)
Bean and Rice Burritos	15 (46.9)	11 (34.4)	3 (9.4)	2 (6.3)	1 (3.1)
Ginger Glazed Mahi Mahi	17 (53.1)	7 (21.9)	6 (18.8)	1 (3.1)	1 (3.1)
Vegetable Stroganoff	11 (34.4)	12 (37.5)	2 (6.3)	5 (15.6)	2 (6.3)
Pasta with Chickpeas, Tomato, and Spinach	10 (34.5)	10 (34.5)	4 (13.8)	3 (10.3)	2 (6.9)
Spaghetti Squash with Meat Sauce	4 (43.8)	8 (25.0)	4 (12.5)	2 (6.3)	4 (12.5)

and Whole Grain Roll	
